# 肺叶切除治疗早期肺癌需要考虑的几个问题

**DOI:** 10.3779/j.issn.1009-3419.2016.06.10

**Published:** 2016-06-20

**Authors:** 俊 王, 辉 赵

**Affiliations:** 100044 北京，北京大学人民医院胸外科 Department of Thoracic Surgery, Peking University People's Hospital, Beijing 100044, China

**Keywords:** 肺肿瘤, 亚肺叶切除, 肺叶切除, Lung neoplasms, Sublobar resection, Lobectomy

## Abstract

随着医学技术的进步，早期肺癌患者数量逐年增加，亚肺叶切除治疗在早期肺癌个体化治疗的价值逐渐受到重视。目前，在早期肺癌的术式选择上，肺叶切除和亚肺叶切除孰优孰劣尚存在一些争议。本文就当前亚肺叶切除治疗早期肺癌的下述争议点进行了总结：①循证医学证据；②楔形和肺段的适应症选择；③肿瘤直径和切缘距离的权重比较；④老年人的术式选择。

## Abstract (en)

With advances in medical technology, theproportion of early-stage lung cancerhas been growing. Conforming to the trend of individualized treatment, sublobar resection for early stage lung cancer has been considered more valuable than ever before. Currently, there were still confilictions about the oncological validity of sublobar resection comparing with lobectomy for lung cancer. We reviewed the study status of the following contentious issues: the evidence based medicine, the indications of wedge resection or segmentectomy, the weight of tumor diameter and margin distance, and the choices for the elderly.

随着CT肺癌筛查的普及，早期肺癌的发现率逐年升高。手术是目前早期非小细胞肺癌最有可能治愈的治疗方式，公认的早期非小细胞肺癌（non-small cell lung cancer, NSCLC）的标准手术方式是根治性肺叶切除加淋巴结清扫。但随着医学技术和治疗理念的进步，近年来肺癌的治疗日趋规范化和个体化，在完整切除肿瘤病灶的前提下，最大限度地保存患者的正常肺组织，减少手术创伤成为外科手术追求的发展方向，对于肺功能差的患者具有更大的现实意义。由此亚肺叶切除治疗早期肺癌的微创优势获得了胸外科学者的重视，相关的研究逐渐升温。本文针对早期肺癌亚肺叶切除的一些焦点问题的研究现状进行综述。

## 1 亚肺叶切除对比肺叶切除的循证医学证据

目前胸外科领域对于早期肺癌的术式选择依旧存在不小的争议。早在1995年Ginsberg领导的美国肺癌研究组（Lung Cancer Study Group, LCSG）完成了迄今唯一一项肺叶切除与亚肺叶切除治疗≤3 cm早期NSCLC的前瞻性多中心随机对照研究。研究结果显示虽然亚肺叶切除术术后远期生存率与肺叶切除相近，但其局部复发率高于肺叶切除术，尤其是肺楔形切除术局部复发率更高，因此仍推荐肺叶切除治疗早期非小细胞肺癌^[1]^。这项研究巩固了肺叶切除在早期肺癌手术中的金标准地位，亚肺叶切除仍旧为高危患者的“妥协”性术式。然而自此20余年来，这项研究在研究结论、研究设计甚至样本量计算等方面广受质疑。最大的争议在于“时过境迁”。这项研究的入组阶段为1982年-1988年，当时尚采用胸片进行术前分期，临床分期的准确性差；研究的入组条件界定于3 cm，“周围型”标准以气管镜界定，这些研究设计在当前看来，无一不是亚肺叶切除局部复发率的高危因素^[2]^。因此，这一研究结果非但未能平复亚肺叶切除与肺叶切除孰优孰劣的话题争议，反而让这一话题成为20余年的临床研究热点。总体而言，从研究结果上看，日本的研究结果多提示亚肺叶切除与肺叶切除的效果相近^[3]^，而美国的大数据结果则更支持肺叶切除的生存优势^[4, 5]^，相似结论是肺癌越早期、患者越高危，两种术式的效果越接近，共性问题是研究证据级别低，研究异质性差，前瞻性研究较少，因此难有说服力^[6]^。目前有两项正在进行的亚肺叶切除前瞻性随机对照临床试验美国CALBG 140503和日本JCOG0802/WJOG4607L，都在比较肿瘤大小≤2 cm的周围型早期肺癌亚肺叶切除和肺叶切除间总生存期、无病生存期、局部和全身复发的风险^[2]^，希望这两项研究能够为亚肺叶切除治疗早期肺癌的地位这一争议话题提供盖棺定论的高水平临床证据。

## 2 楔形切除还是肺段切除？

亚肺叶切除包括楔形切除与亚肺叶切除，两种亚肺叶切除术式的适应证选择，LCSG的前瞻性研究楔形切除比例过高就曾受到过质疑，在临床上并无定论。一般而言，肺段切除与楔形相比，其优势在于依照肺癌引流区域的解剖性切除和更为全面的淋巴结评估，但其劣势是手术相对复杂，并发症风险更高。楔形切除与肺段切除的远期效果比较，目前存在的争议较多，缺少足够的研究。一项美国SEER数据库的大样本回顾性分析，对3, 525例Ⅰa期NSCLC行亚肺叶切除患者的生存资料进行了分析，发现肺段切除术总生存率和肺癌特异性生存率均明显优于肺楔形切除术，且这种生存优势在根据病变性质、肿瘤最大径≤2 cm和年龄 > 70岁等分层因素进行匹配后依然存在^[7]^。2014年Reveliotis等^[8]^回顾过去25年间的45个临床研究，认为肺段切除术的局部复发率和肿瘤相关死亡率均优于肺楔形切除，建议行肺段切除。总体而言，目前更多的证据提示肺段切除术治疗早期肺癌疗效优于楔形切除，虽然这些研究的证据数量和质量还不充分。然而，临床上开展亚肺叶切除手术时，应如何选择两种手术的适应证，至少目前鲜有证据。这将成亚肺叶切除治疗早期肺癌的一个焦点问题。

## 3 肿瘤直径还是切缘距离

1994年Warren^[9]^首先发现亚肺叶切除对于直径 < 2 cm肺癌的治疗效果更佳，提示肿瘤直径是影响亚肺叶切除的治疗效果的重要预后因素。后续研究关于亚肺叶切除的适应征选择更为严谨，集中在直径 < 2 cm的早期肺癌。多项对照研究也证实直径 < 2 cm的早期肺癌行肺叶和亚肺叶切除的生存率并无统计学差异。因此，直径 < 2 cm成为现阶段亚肺叶切除手术适应证的最重要指标。对于更大直径（2 cm-3 cm）的早期肺癌，目前的临床研究存在着争议，Nakamura^[10]^的*meta*分析和Schuchert^[11]^的回顾性分析结果提示对于Ⅰ期肺癌行亚肺叶切除生存率较肺叶切除并未降低。Okada的研究虽然指出了对于2 cm-3 cm的肺癌，肺叶对于亚肺叶组存在生存优势，但根据其结果肺叶组和肺段组并无差异，差异主要体现在楔形组^[12]^。因此，直径2 cm-3 cm的早期肺癌，一般不选择亚肺叶切除手术。

但实际上，有证据显示切缘距离才是影响亚肺叶切除手术复发、转移的根本原因。早在2007年，El-Sherif等^[13]^便提出亚肺叶切除保留足够切缘距离的重要性，作者回顾了81例接受亚肺叶切除（楔形及肺段）的早期NSCLC患者资料，其中41例切缘 < 1 cm、40例切缘 > 1 cm。在对无病生存和局部复发的分析中两组表现出统计学差异。切缘小于1 cm中14.6%（6/41）发生了局灶复发，而大于1 cm中7.5%（3/40）复发。2014年Mohiuddin等^[14]^的回顾性研究发现，对于最大径≤2 cm的NSCLC，切缘增加到10 mm相对于5 mm切缘能降低45%的局部复发率，15 mm的切缘似是最佳距离，切缘继续增加并未使局部复发率继续降低。但是，2015年Maurizi等^[15]^的研究认为，不论切缘距离是否小于1 cm，介于1 cm和2 cm，以及大于2 cm，在疾病的复发和总的生存率均没有统计学差异。目前采用 > 1 cm切缘的综述分别发表在权威的《*Semin Thorac Cardiovasc Surg*》和《*Eur Respir J*》杂志上；肿瘤最大径和≥2 cm标准则分别被CALGB140503、JCOG0802研究和NCCN非小细胞肺癌治疗指引采用。

然而，新的研究提示亚肺叶切除复发转移不仅仅与直径和切缘有关。2015年Kadota^[16]^对于肺癌气腔传播的回顾性研究显示，直径≤2 cm的Ⅰ期肺癌中，38%存在气腔传播，气腔传播是局限性切除术后复发的风险因素。这一研究给我们提出了新的警示，如何权重肿瘤直径、切缘距离，如何评估气腔传播，已成为亚肺叶切除治疗早期肺癌的一个焦点问题。

## 4 老年人早期肺癌是亚肺叶切除的优选人群？

老年人群是肺癌的好发人群。美国监测、流行病学与最终结果数据库统计数据显示，2005年-2009年间，70岁以上患者比例已高达71%，其中三分之一超过80岁。对于早期NSCLC，手术切除是首选治疗，标准手术方式是肺叶切除。但是，高龄老年患者沿用这些来自年轻患者研究的原则，带来了各种问题。即便是顶级的医疗机构Mayo Clinic，老年患者肺叶切除的手术并发症发生率也高达48%，死亡率6.3%^[17]^。

对于手术风险相对较高的老年患者，包括肺段切除和楔形切除在内的“妥协性”的亚肺叶切除可能有限地降低手术风险。匹兹堡大学医学中心Kilic等^[18]^的研究则提示对于75岁以上的Ⅰ期高龄老年NSCLC患者，肺段切除的生存率与肺叶切除相近，且围术期并发症发生率降低（29.5% *vs* 50%），但局部复发率和总生存情况与肺叶切除类似。

对于预期寿命有限的老年患者，随着年龄的增加，亚肺叶切除与肺叶切除对于早期NSCLC的远期疗效更加接近。SEER数据库14, 555例Ⅰ期和Ⅱ期NSCLC的回顾性分析发现，肺叶切除相比亚肺叶切除的生存优势仅局限于71岁以下人群，而在71岁以上人群，肺叶切除与亚肺叶切除的生存率并无统计学差异^[19]^。来自日本的回顾性研究也发现，75岁以下Ⅰa期NSCLC术后5年生存率亚肺叶切除组低于肺叶切除术组（平均年龄68岁*vs* 64岁，5年生存率64.0% *vs* 90.9%），但在75岁以上患者中两种术式之间的生存率变得更为接近（平均年龄78岁*vs* 77岁，5年生存率67.6% *vs* 74.3%），不再有统计学差异^[20]^。所以亚肺叶切除术式可能是老年早期肺癌最为恰当的根治性手术方式。

为了明确亚肺叶切除在老年早期肺癌患者中的治疗地位，我中心发起了一项“亚肺叶切除对比肺叶切除治疗≥70岁cT1N0M0期NSCLC的多中心、随机、开放Ⅲ期临床试验”（STEPS研究）^[21]^。这是首个专注于老年患者这一肺癌主体人群的外科临床试验。我们希望通过这一研究，明确亚肺叶切除手术在老年早期肺癌患者的近期和远期效果，实现老年肺癌手术患者的生存时间与生活质量的最大获益。
